# Study Protocol and Characteristics of the Wearable Device Survey for Tohoku Medical Megabank Brain Magnetic Resonance Imaging Study

**DOI:** 10.2188/jea.JE20250540

**Published:** 2026-11-05

**Authors:** Ippei Chiba, Naoki Nakaya, Shunji Mugikura, Mana Kogure, Rieko Hatanaka, Kumi Nakaya, Sayuri Tokioka, Masato Takase, Tomohiro Nakamura, Satoshi Nagaie, Soichi Ogishima, Taku Obara, Ikuko N. Motoike, Naoko Mori, Hisanori Matsui, Tatsuya Ando, Hideki Kobayashi, Taketoshi Ogawa, Shinji Tanaka, Nobuo Fuse, Fuji Nagami, Yoko Izumi, Shinichi Kuriyama, Atsushi Hozawa

**Affiliations:** 1 Tohoku Medical Megabank Organization, Tohoku University, Sendai, Japan; 2 Graduate School of Medicine, Tohoku University, Sendai, Japan; 3 Faculty of Data Science, Kyoto Women’s University, Kyoto, Japan; 4 Advanced Research Center for Innovations in Next-Generation Medicine, Tohoku University, Sendai, Japan; 5 Graduate School of Medicine, Akita University, Akita, Japan; 6 Research, Takeda Pharmaceutical Company Limited, Kanagawa, Japan; 7 R&D Division, Daiichi Sankyo Co., Ltd., Tokyo, Japan; 8 International Institute of Disaster Science, Tohoku University, Sendai, Japan

**Keywords:** wearable device, physical activity, sleep, brain health

## Abstract

Sleep and physical activity play a crucial role in brain and mental health. While traditional self-reported methods and research-grade accelerometers have several limitations, consumer-grade wearable devices, such as Fitbit, allow continuous, objective, and real-life data collection. The purpose of this article is to report the study protocol and participant characteristics of a wearable device survey that primarily aims to examine the relationship between brain health and long-term patterns of daily physical activity and sleep. Nearly 2,000 participants were recruited between September 2022 and December 2023, as part of the Tohoku Medical Megabank Brain Magnetic Resonance Imaging Study, to wear Fitbit Charge 5 devices for 1 year, continuously tracking physical activity and sleep. Additionally, home blood pressure measurements and questionnaire data on housing conditions, psychological distress, and medication were collected every 4 months for a total of four assessments. The mean age of the participants was 58.5 years and 37.4% were men. For the first 30 days after the recruitment, mean step count was 8,910 steps/day, and the mean total sleep time was 370.4 minutes/night (approximately 6.2 hours). The mean morning systolic blood pressure and diastolic blood pressure were 126.2 and 75.5 mm Hg, respectively. This study provides a unique opportunity to integrate longitudinal consumer-grade wearable data, brain MRI, multiomics data, and comprehensive health records from existing cohorts to advance precision medicine and help prevent mental and neurodegenerative diseases, such as dementia. Limitations include potential selection bias, and a relatively smaller sample size than larger global studies.

## PURPOSE

Low sleep quality and insufficient physical activity are globally prevalent,^1^^,^^2^ threatening public health. Both low sleep quality and physical inactivity have been linked to several non-communicable diseases, including dementia.^3^^–^^6^ Such evidence indicates that management of sleep and physical activity promotes healthy longevity in ageing societies. Monitoring these behaviors is helpful to maintain brain health. For instance, sleep condition and its pattern can predict mood disorders^7^^–^^9^ and dementia^10^^,^^11^; physical activity monitoring can predict dementia.^12^^,^^13^ Importantly, sleep and physical activity interact^14^^,^^15^ and fluctuate over time^16^^,^^17^; fragmented assessments fail to capture their complex associations to health. Therefore, simultaneous and longitudinal measurements are essential for understanding their dynamics and long-term impacts.

Traditionally, sleep and physical activity have been assessed using self-reported measures, which are subject to recall bias^18^^,^^19^ and correlate poorly with objective indicators.^20^^,^^21^ Self-reporting also requires participant effort and is generally unsuitable for continuous, long-term data collection. Research-grade accelerometers, such as ActiGraph^22^ (used in several studies) and Axivity AX3^23^ (used in the UK Biobank), can provide detailed objective data, but their use is often limited by short monitoring duration due to internal memory constraints. In recent years, the widespread adoption of consumer-grade wearable devices has enabled the collection of continuous real-life personal health records (PHRs). These devices can track multiple physiological parameters with reasonable reliability, including step counts, activity intensity, sleep duration (eg, total sleep time), and heart rate, by syncing data to cloud servers rather than relying on internal memory. Putative sleep stages (eg, deep, rapid eye movement [REM], and light sleep) can also be algorithmically inferred. As such, they offer a feasible solution for long-term, concurrent monitoring of daily behaviors. Crucially, the potential of these PHRs can be maximized when integrated with detailed clinical phenotyping. Combining multi-dimensional health measures, including associations of brain structure with function, allows for a comprehensive understanding of healthy longevity. However, few studies have systematically established a cohort that integrates such long-term wearable device data with these data.

The purpose of this article is to report the study protocol and participant characteristics, including long-term daily physical activity and sleep conditions, from the wearable device survey designed to be integrated with the brain MRI data and multi-dimensional health record data from the Tohoku Medical Megabank (TMM) Project. Establishing this baseline is a critical step toward utilizing this comprehensive dataset to elucidate the complex relationships between daily lifestyle behaviors and healthy longevity.

## MAIN FEATURES

In 2022, we initiated a wearable device sub-study within the Tohoku Medical Megabank Brain Magnetic Resonance Imaging Study (TMM Brain MRI Study).^24^ This sub-study consisted of 1 year of continuous tracking of daily physical activity and sleep using Fitbit devices, which have demonstrated validity for both measurements.^25^^–^^28^ Nearly 2,000 participants were asked to wear a Fitbit Charge 5 (Fitbit, Inc., San Francisco, CA, USA) for 1 year, continuously throughout the day except while bathing. All participants were supplied with Fitbit devices and a manual. They also received instructions from trained staff on how to use the device. Fitbit application was installed on each participant’s smartphone. Participants were also instructed to interrupt wearing the Fitbit device if they experienced any adverse physical (eg, skin irritation) or psychological event (eg, insomnia) until the condition was resolved. Additionally, home blood pressure, questionnaire responses, and housing temperature and humidity, which were recorded for only 180 participants due to equipment limitations, were measured during four 1-month intensive measurement periods. These periods occurred every 4 months following participant recruitment (Figure 1). All participants received a 500 Japanese yen voucher as an incentive for each of the intensive measurement periods.

**Figure 1.**
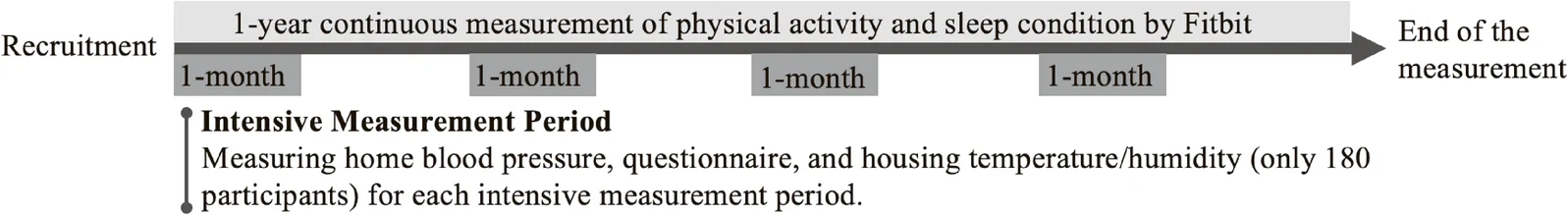
Measurement protocol for the study

### Participants

Participants in this survey were recruited from the secondary survey (October 2019 to March 2024) of the TMM Brain MRI Study.^24^ This ongoing study has repeatedly measured brain structure, cognitive function, and psychological status since 2014, with its third survey currently underway in 2025. The TMM Brain MRI Study comprises a subset of participants aged 20 years or older from both the Tohoku Medical Megabank Community-Based Cohort Study (TMM CommCohort Study)^29^^,^^30^ and the Tohoku Medical Megabank Birth and Three-Generation Cohort Study (TMM BirThree Cohort Study),^31^^,^^32^ both of which include detailed lifestyle information, physiological measurements, and multiomics data (eg, metabolite and genomic data).

We recruited 2,000 participants based on the sample size of previous research that conducted a genome-wide association analysis of device-measured sleep^33^ and on the number of participants in the TMM Brain MRI Study.^24^ We recruited participants between September 2022 and December 2023 using two procedures to achieve the planned sample size during the secondary survey of the TMM Brain MRI Study.^24^ (1) Concurrent-visit recruitment: participants were invited to join this survey during their secondary visit to the TMM Brain MRI Study. (2) Post-visit recruitment: we mailed invitations to individuals who had participated in the TMM Brain MRI Study before the survey’s launch (eFigure 1). Finally, 1,996 participants were included in the survey: 1,105 from the concurrent-visit recruitment and 891 from the post-visit recruitment (Figure 2). Individuals who did not have smartphones (including incompatible devices with Fitbit application) were excluded. Regarding the non-smartphone owners, 70.0% were aged over 70 years, and 48.5% were female. Exclusions based on smartphone ownership occurred only in concurrent-visit recruitment. For post-visit recruitment, we explicitly asked potential targets to ensure their devices supported the Fitbit application before applying, resulting in no further exclusions for this reason.

**Figure 2.**
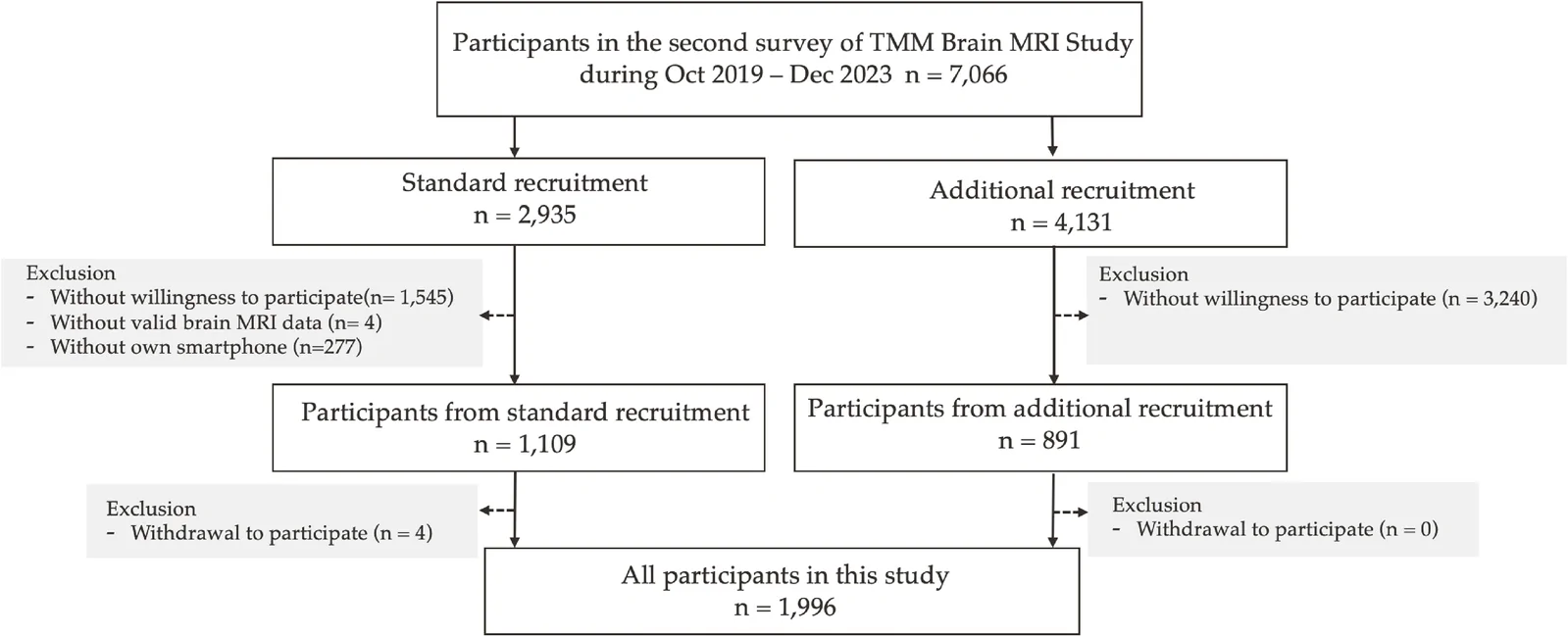
Flow diagram of recruitment

All participants provided written informed consent to participate in the study. This study was approved by the Institutional Review Board of the Tohoku Medical Megabank Organization (approval number: 2021-1021).

## MEASUREMENTS OF THE PRESENT STUDY

### Device measured physical activity and sleep condition

Data from the Fitbit Charge 5 were collected via the Fitbit Web API.^34^ Specifically, we obtained sleep data from “Get Sleep Logs,” physical activity data (excluding global positioning data) from “Get Daily Activity Summary,” heart rate data from “Get Heart Rate Time Series,” and participant characteristic data from “Get Profile.” We also accessed the “Get Heart Rate Intraday Time Series” to calculate device-wearing time. The Fitbit Charge 5 measured the heart rate via an optical sensor in 5-second epochs. We summed all the epochs in which the heart rate was measured to determine the total daily wearing time. Ultimately, we collected the following data: (1) sleep data, including total and stage-specific (deep, REM, or light) daily sleep time and sleep efficiency at both daily and intraday levels; (2) physical activity data, including steps, intensity-specific physical activity time (sedentary, lightly active, fairly active, and very active), and estimated daily energy expenditure; (3) heart rate data, including resting heart rate and estimated device wearing time; and (4) profile data, including self-reported age, sex, height, and body weight. Detailed information on the data from the Fitbit Web API has been described elsewhere.^34^ To ensure participant privacy, study-specific IDs were used for cloud-based data acquisition, excluding both personally identifiable information and global positioning system location data.

### Home blood pressure

Home blood pressure was measured using an electronic upper arm cuff device (HEM-7080IC; OMRON Healthcare, Kyoto, Japan). The participants were asked to measure their morning and evening blood pressure daily during each intensive measurement period. Consistent with Japanese guidelines,^35^ morning blood pressure was measured while seated, within 1 hour of awakening, after 1–2 minutes of rest, and before breakfast or medication intake. Blood pressure was measured 1–2 minutes after sitting, just before bedtime.

### Other measurements

The participants were asked to complete the questionnaire during the intensive measurement period. The questionnaire included the following: (1) housing conditions, such as coldness, warmth, and safety, using the Comprehensive Assessment System for Building Environment Efficiency (CASBEE) checklist^36^; (2) self-reported sleep time and condition using the Japanese version of the Athens Insomnia Scale (AIS-J)^37^^,^^38^; (3) psychological distress using the Kessler 6-item Psychological Distress Scale (K6)^39^^,^^40^; and (4) medications, dietary supplements, and health foods.

A subset of participants (*n* = 180) measured objective bedroom conditions using a temperature and humidity logger (TR-72nw; T&D Corp, Nagano, Japan), which recorded these conditions every 15 minutes, during every intensive measurement period. We asked participants to set up the logger in their bedroom, positioning it at least 1 m above the floor.

All variables collected in this study are shown in eTable 1.

## EXISTING COHORT DATA FROM TMM PROJECT

### Characteristics and outcome and follow-up

Variables characterizing the participants were gathered from the existing cohort of TMM Project. For instance, baseline brain structure and cognitive function data were collected in the TMM brain MRI study,^24^ and questionnaire-based socioeconomic status (SES), lifestyle and comorbidities, physiological data, and laboratory data were measured in the TMM CommCohort^29^^,^^30^ and BirThree Cohort Study.^31^^,^^32^ Outcomes of the present study were also collected in these existing cohorts: the incidence of several diseases, such as neurodegenerative and mental disorders were collected from the TMM CommCohort^30^ and BirThree Cohort Study^32^; and repeatedly measured brain structure and function data were collected from the TMM Brain MRI Study.^24^

## BASELINE CHARACTERISTICS

### Data processing of device-measured physical activity, sleep, and home blood pressure data

This study presented the baseline characteristics of device-measured sleep, physical activity, and home blood pressure data. Prior to analysis, we operationally processed the data as follows: for sleep data, we included only the main sleep records, as defined by the Fitbit device, which allowed calculation of stage-specific (deep, REM, or light sleep) daily sleep time, thereby excluding naps. Sleep records were included if the total sleep duration ranged from 3 to 12 hours and if the sleep onset time fell between 20:00 and 02:00.^41^^,^^42^ Furthermore, valid data meeting these conditions were available for at least 50% of weekdays (including the previous day) and weekends/holidays throughout the measurement period. For the physical activity data, we only included records with a wear time of at least 10 hours per day and a daily step count between 1,000 and 45,000 steps.^43^ Additionally, valid data meeting these criteria were available for at least 50% of weekdays and weekends/holidays during the measurement period. Sedentary time was calculated as the residual of total wearing time minus the time spent in bed rest, sleep (as defined by Fitbit sleep data), and light- or higher-intensity physical activity. For home blood pressure measurements, we identified measurements from 03:00 to 10:00 as the morning blood pressure and from 18:00 to 24:00 as the evening blood pressure. Valid morning and evening blood pressure data meeting these criteria were available for at least 50% of weekdays and weekends/holidays during the measurement period. These criteria did not fully correspond to those used in previous studies.

Baseline characteristics, including basic participant data, device-measured sleep and physical activity data, and home blood pressure, were derived from summary statistics collected during the first 30 days after participation. These characteristics are presented for all participants overall and for each age category group (20–39, 40–49, 50–59, 60–69, 70–79, and 80 years and older). The longitudinal trends for each data type are displayed as monthly summary statistics.

### Participants’ demographic data and adherence

The mean age of the participants was 58.5 (standard deviation [SD], 13.9) years, and 37.4% were men. The monthly mean device wear time exceeded 1,200–1,300 minutes per day for person-day data. Approximately 80% of the person-day time data indicated a wear time of >20 hours per day.

### Physical activity

During the baseline period, 1,892 participants provided valid physical activity data. For all participants, the mean step count was 8,910 (SD, 3,382) steps per day; sedentary time was 689.6 (SD, 124.9) minutes/day; light intensity physical activity (LPA), using the “light active” Fitbit parameter, was 271.3 (SD, 79.2) minutes/day; and moderate-to-vigorous physical activity (MVPA), using the “fairly active” and “very active” Fitbit parameters, was 33.0 (SD, 30.5) minutes/day. Older age groups (70–79 and ≥80 years) had a higher proportion of males (52.6% for 70–79 years and 62.7% for ≥80 years) than younger age groups (24.7–36.4%). While the MVPA time was higher in participants aged 60 years or older, the LPA time was lower in this group (Table 1). eFigure 2 shows age-stratified distribution of the physical activity data. Characteristics of physical activity variables stratified by recruitment category (concurrent-visit or post-visit) are shown in eTable 2. For overall period, the monthly mean step count varied between 8,000 and 10,000 steps/day; LPA time ranged from 250 to 300 minutes/day; MVPA varied from 20 to 40 minutes/day; and sedentary time ranged from 500 to 700 minutes/day. Figure 3 shows the monthly mean (solid line) and standard deviation (shaded area) of the monthly trends in physical activity data for overall period. Detailed characteristics of these data are also shown in eTable 3.

**Figure 3.**
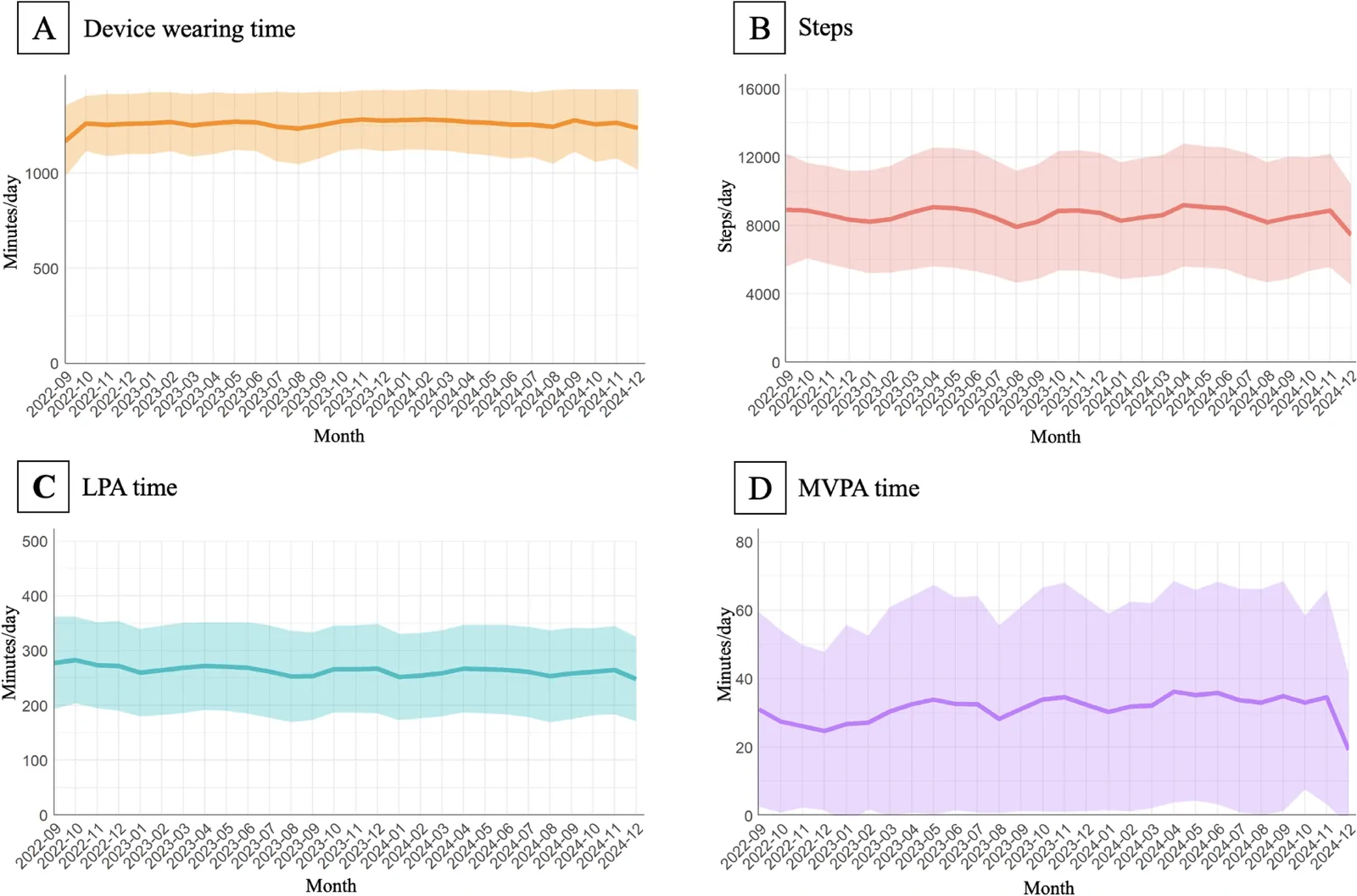
Monthly trends in physical activity data for overall period. The plots show (**A**) device wearing time (minutes/day), (**B**) daily step counts, (**C**) light physical activity (LPA) time (minutes/day), and (**D**) moderate-to-vigorous physical activity (MVPA) time (minutes/day) measured by Fitbit from September 2022 to December 2024. Solid lines represent the monthly mean values, and the shaded areas indicate standard deviation.

**Table 1.** Characteristics of baseline physical activity-associated variables

Variables	Overall	Age category, years					

		20–39	40–49	50–59	60–69	70–79	≥80
*N*	1,892	201	428	295	420	473	75
Age, years, mean (SD)	58.5 (13.8)	36.3 (2.5)	44.7 (2.8)	54.8 (2.9)	64.7 (2.8)	73.6 (2.4)	81.6 (2.3)
Sex, men, *n* (%)	700 (37.0%)	57 (28.4%)	156 (36.4%)	73 (24.7%)	118 (28.1%)	249 (52.6%)	47 (62.7%)
Device wearing time, min/day, mean (SD)	1,290.2 (121.9)	1,271.0 (135.9)	1,273.2 (135.4)	1,290.8 (128.1)	1,306.4 (105.3)	1,297.8 (112.5)	1,297.4 (103.4)
Steps, steps/day, mean (SD)	8,910 (3,382)	8,773 (3,163)	8,995 (3,229)	9,264 (3,138)	9,158 (3,569)	8,704 (3,525)	7,304 (3,268)
Sedentary time, min/day, mean (SD)	689.6 (124.9)	694.1 (123.5)	702.2 (121.8)	695.9 (116.7)	672.8 (122.9)	681.9 (131.8)	722.7 (131.5)
LPA time, min/day, mean (SD)	271.3 (79.2)	286.5 (73.1)	280.4 (75.5)	293.0 (81.9)	283.4 (77.4)	242.9 (73.2)	205.6 (68.6)
MVPA time, min/day, mean (SD)	33.0 (30.5)	25.6 (25.3)	30.1 (30.8)	27.4 (24.6)	32.6 (29.6)	41.8 (34.4)	38.4 (28.8)
Season at recruitment							
Autumn, *n* (%)	675 (35.7%)	61 (30.3%)	125 (29.2%)	114 (38.6%)	171 (40.7%)	177 (37.4%)	27 (36.0%)
Spring, *n* (%)	442 (23.4%)	52 (25.9%)	96 (22.4%)	63 (21.4%)	88 (21.0%)	117 (24.7%)	26 (34.7%)
Summer, *n* (%)	541 (28.6%)	62 (30.8%)	142 (33.2%)	81 (27.5%)	128 (30.5%)	113 (23.9%)	15 (20.0%)
Winter, *n* (%)	234 (12.4%)	26 (12.9%)	65 (15.2%)	37 (12.5%)	33 (7.9%)	66 (14.0%)	7 (9.3%)

LPA, light intensity physical activity; MVPA, moderate-to-vigorous intensity physical activity; SD, standard deviation.

### Sleep condition

During the baseline period, 1,559 participants provided valid sleep data. For all participants, the mean total, deep, REM, and light sleep times were 370.4 (SD, 42.1), 58.1 (SD, 13.1), 76.4 (SD, 18.3), and 235.9 (SD, 36.2) minutes/night, respectively. The mean sleep efficiency was 93.4% (SD, 4.3%). Total sleep time was higher in the older age group, but deep and REM sleep times were lower (Table 2). eFigure 3 shows age-stratified distribution of the sleep data. Characteristics of sleep variables stratified by recruitment category (concurrent-visit or post-visit) are shown in eTable 4. For overall period, the monthly mean total sleep time ranged from 350 to 400 minutes/night, deep sleep from 55 to 65 minutes/night, REM sleep from 70 to 85 minutes/night, and light sleep from 200 to 250 minutes/night. Figure 4 shows the monthly mean (solid line) and standard deviation (shaded area) of the monthly trends in sleep data for overall period. Detailed characteristics of these data are also shown in eTable 5.

**Figure 4.**
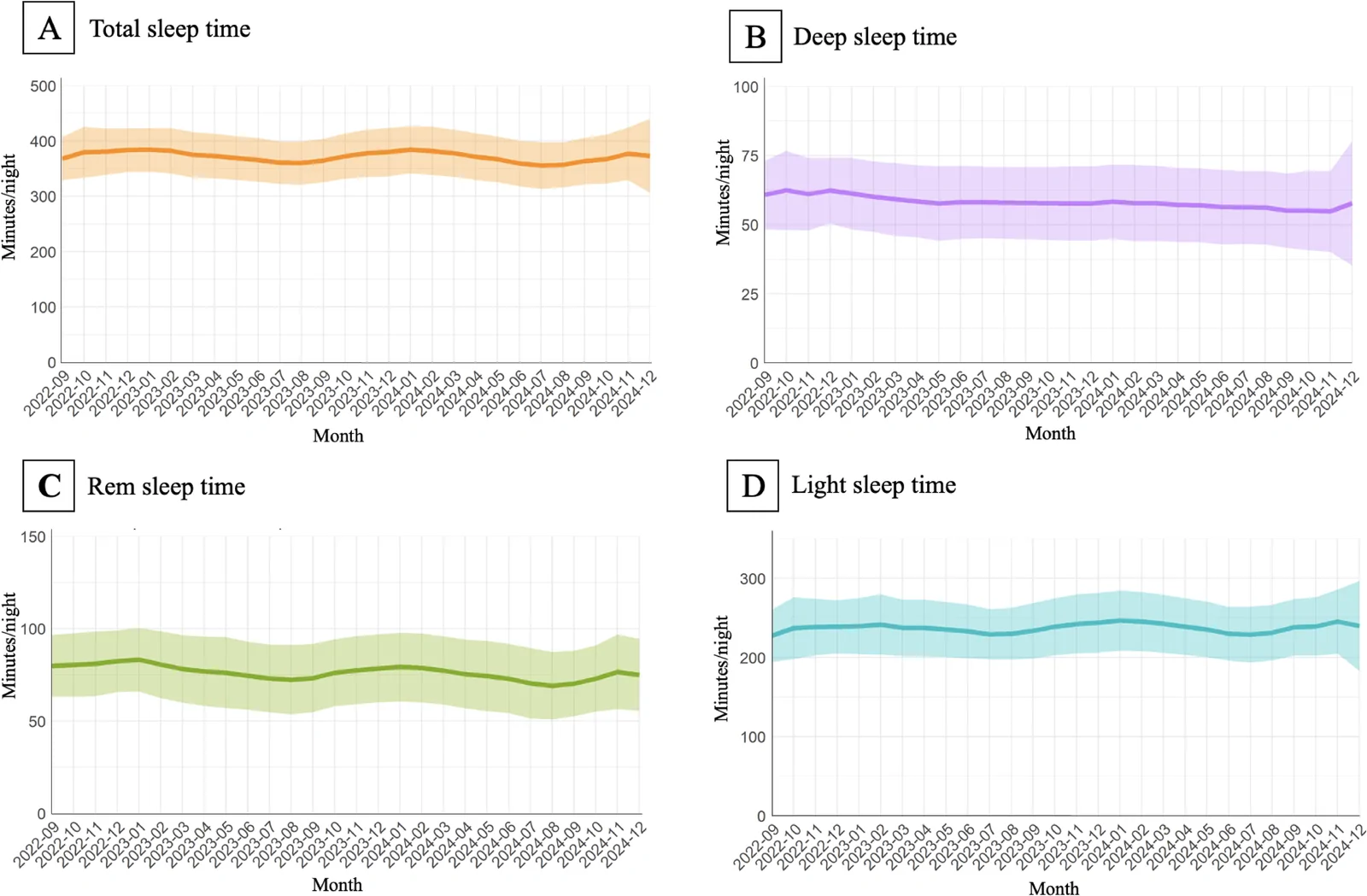
Monthly trends in sleep data for overall period. The plots illustrate (**A**) total sleep time, (**B**) deep sleep time, (**C**) REM sleep time, and (**D**) light sleep time (all in minutes/night) measured by Fitbit from September 2022 to December 2024. Solid lines represent the monthly mean values, and the shaded areas indicate standard deviation.

**Table 2.** Characteristics of baseline sleep-associated variables

Variables	Overall	Age category, years					

		20–39	40–49	50–59	60–69	70–79	≥80
*N*	1,559	175	354	242	366	376	46
Age, years, mean (SD)	58.0 (13.7)	36.4 (2.3)	44.5 (2.9)	54.7 (2.9)	64.7 (2.8)	73.4 (2.4)	81.5 (1.9)
Sex, men, *n* (%)	543 (34.8%)	53 (30.3%)	124 (35.0%)	58 (24.0%)	101 (27.6%)	181 (48.1%)	26 (56.5%)
Time in bed, min/night, mean (SD)	426.8 (49.0)	433.4 (50.9)	417.1 (45.1)	417.8 (46.3)	424.8 (47.0)	436.9 (50.5)	457.3 (56.4)
Deep sleep time, min/night, mean (SD)	58.1 (13.1)	67.1 (11.6)	62.7 (11.7)	59.2 (12.0)	55.6 (12.2)	52.2 (12.6)	51.2 (13.3)
REM sleep time, min/night, mean (SD)	76.4 (18.3)	85.8 (17.8)	81.4 (15.7)	78.3 (16.4)	74.3 (18.3)	68.8 (18.4)	70.1 (20.0)
Light sleep time, min/night, mean (SD)	235.9 (36.2)	226.1 (34.4)	220.8 (28.5)	225.5 (31.7)	237.3 (33.3)	255.4 (37.2)	272.9 (35.4)
Total sleep time, min/night, mean (SD)	370.4 (42.1)	379.1 (44.9)	364.9 (38.8)	363.0 (39.2)	367.2 (40.5)	376.4 (43.9)	394.1 (49.8)
Sleep efficiency, %/night, mean (SD)	93.4 (4.3)	93.7 (5.4)	93.6 (6.4)	93.6 (4.1)	93.5 (2.9)	93.1 (2.6)	93.1 (1.9)
Season at recruitment							
Autumn, *n* (%)	574 (36.8%)	52 (29.7%)	106 (29.9%)	101 (41.7%)	149 (40.7%)	145 (38.6%)	21 (45.7%)
Spring, *n* (%)	357 (22.9%)	46 (26.3%)	78 (22.0%)	48 (19.8%)	77 (21.0%)	94 (25.0%)	14 (30.4%)
Summer, *n* (%)	440 (28.2%)	53 (30.3%)	117 (33.1%)	62 (25.6%)	111 (30.3%)	90 (23.9%)	7 (15.2%)
Winter, *n* (%)	188 (12.1%)	24 (13.7%)	53 (15.0%)	31 (12.8%)	29 (7.9%)	47 (12.5%)	4 (8.7%)

REM, rapid eye movement; SD, standard deviation.

### Home blood pressure data

During the baseline period, 1,908 participants provided valid home blood pressure data. For all participants, the mean morning systolic blood pressure (SBP), morning diastolic blood pressure (DBP), evening SBP, and evening DBP values were 126.2, 75.5, 122.1, and 71.2 mm Hg, respectively. The proportions of participants with morning and evening hypertension, defined by a SBP of 135 mm Hg or higher or DBP of 85 mm Hg or higher irrespective of medication status, were 30.9% and 18.2%, respectively. Both morning and evening SBP were higher in older participants. Conversely, both morning and evening DBP values were higher in the middle-to-older age groups (50–69 years) but lower in the younger and oldest age groups.

### Characteristics from existing cohort

eTable 6 shows the characteristics of potential targets (the participants of the secondary survey of TMM brain MRI study^24^), stratified by participants (*n* = 1,996) and non-participants (*n* = 5,444). Variables include cognitive function from the secondary survey of TMM brain MRI study,^24^ SES, questionnaire-based lifestyle and comorbidity data, physiological data, and laboratory data from the baseline and secondary survey of the TMM CommCohort^29^^,^^30^ and BirThree Cohort Study.^31^^,^^32^ The participants of the present study were younger (57.6; SD, 13.8 years for participants and 60.2; SD, 14.4 years for non-participants) and tended to have higher educational attainment (university or higher education: 43.0% for participants and 36.3% for non-participants) than non-participants.

## STRENGTHS AND LIMITATIONS

Our study has several strengths. First, to the best of our knowledge, this is the first study to measure longitudinal PHRs from consumer-grade devices alongside brain MRI and cognitive assessments. For example, although the All of Us program^44^^,^^45^ collected Fitbit data longitudinally, including periods prior to recruitment,^46^ detailed brain structures and cognitive functions have not yet been measured in that initiative. Similarly, in the UK Biobank study, both brain MRI and accelerometer-measured physical activity and sleep conditions were collected^47^; however, the measurements were not continuous (only 7 days) despite the fact that sleep and physical activity show seasonal variety and fluctuation.^16^^,^^17^ Compared with these large cohort studies that include objective physical activity and sleep data, our study offers a unique opportunity to examine brain structure and longitudinal changes in physical activity and sleep conditions. Second, the baseline values of the Fitbit data were in line with other surveys. Our study revealed that participants averaged 8,910 steps per day, and mean MVPA was 33.0 minutes per day (231.0 minutes per week). For comparison, studies from the All of Us program, which included participants aged 52–56 years, reported a mean of 7,731.3 steps per day^48^ and 247 minutes of weekly MVPA.^22^ The physical activity values observed are largely consistent with those reported in the All of Us study. Our study showed that the mean total sleep time, percentage of deep sleep, percentage of REM sleep, and percentage of light sleep were 370.4 minutes per sleep (6.2 hours), 15.6%, 20.6%, and 63.6%, respectively. These values are largely in line with findings from the All of Us Research program (15.2%, 20.7%, 64.2%, respectively).^42^ Therefore, the physical activity and sleep variables in our study demonstrated validity when compared with other research that had utilized Fitbit devices. Third, this study combined detailed health condition data and multiomics data from the TMM CommCohort and BirThree Cohort Studies. This integration of comprehensive cohort data can promote precision medicine and prevention.

This study has some limitations. First, our study may be subject to selection bias, as participants tended to have a greater interest in health and better health conditions than non-participants. This interpretation is supported by the characteristics comparison between participants and non-participants of the present study (relatively younger and higher educational attainment) and a previous study showing that individuals participating in accelerometer research had superior health compared to those who did not.^49^ In addition, a digital divide may exist, as our study excluded individuals who did not have or could not use digital devices, potentially introducing a selection bias. Second, the total sample size was smaller than that of the Fitbit study (Bring Your Own Device Program and Enhancing All of Us Research) within the All of Us Research Program (*n* = 30,445).^50^

## SUPPLEMENTARY MATERIAL

eTable 1. List of data collected for this study

eTable 2. Characteristics of baseline physical activity-associated variables divided by recruitment category

eTable 3. Characteristics of physical activity-associated variables for overall period

eTable 4. Characteristics of baseline sleep-associated variables divided by recruitment category

eTable 5. Characteristics of sleep-associated variables for overall period

eTable 6. Characteristics of the existing cohort data for the participants and non-participants of the present study

eFigure 1. Timeline of the TMM Brain MRI Study and recruitment for the present study

eFigure 2. Ridgeline plot for baseline physical activity-associated variables stratified by age category

eFigure 3. Ridgeline plot for baseline sleep-associated variables stratified by age category
