# Supplementary material for: Study Protocol and Characteristics of the Wearable Device Survey for Tohoku Medical Megabank Brain Magnetic Resonance Imaging Study
Source: J Epidemiol. 2026 Nov 5;36(11):365–72. doi: 10.2188/jea.JE20250540 (PMC13662269; doi:10.2188/jea.JE20250540)
Supplement: Supplementary file 1 [file je-36-365-s001.pdf]

eTable 1. List of data collected for this study

Data category	Equipment	Data types	Variable examples	Data unit	Measurement period
Fitbit data	Fitbit Charge 5	Heart rate	Resting heart rate	Daily	1 year
			Device-wearing time	Daily	1 year
		Physical Activity	Step counts	Daily	1 year
			Energy expenditure	Daily	1 year
			Intensity-specific physical activity (sedentary, light active, fairly active, and very active) time	Daily	1 year
		Sleep	Sleep duration	Daily, intraday	1 year
			Sleep efficiency	Daily	1 year
			Stage-specific sleep duration (deep, REM, light)	Daily, intraday	1 year
Blood Pressure	HEM-7080IC	Blood Pressure	Morning and evening systolic, diastolic blood pressure, and heart rate	Daily	During each intensive measurement period
Questionnaire	Paper form	Housing condition	Comprehensive Assessment System for Building Environment Efficiency checklist	Per survey	Once per intensive measurement period
		Sleep condition	Athens Insomnia Scale	Per survey	Once per intensive measurement period
		Psychological Distress	Kessler 6 scale	Per survey	Once per intensive measurement period
		Medications	Self-reported medications, dietary supplements, and health foods	Per survey	Once per intensive measurement period
Bedroom condition[a]	TR-72nw	Temperature	Temperature	15 min	During each intensive measurement period
		Humidity	Humidity	15 min	During each intensive measurement period

REM, rapid eye movement.

[a] Only 180 participants were asked to measure the bedroom condition.

eTable 2. Characteristics of baseline physical activity-associated variables divided by recruitment category

Variables	Recruitment category: Concurrent-visit (n=1,033)	Recruitment category: Post-visit (n=859)
Age, years, mean (SD)	57.9 (14.3)	59.0 (13.2)
Age category		
20−39, n (%)	121 (11.7)	80 (9.3)
40−49, n (%)	260 (25.2)	168 (19.6)
50−59, n (%)	144 (13.9)	151 (17.6)
60−69, n (%)	191 (18.5)	229 (26.7)
70−79, n (%)	275 (26.6)	198 (23.1)
≥80, n (%)	42 (4.1)	33 (3.8)
Sex, men, n (%)	403 (39.0)	297 (34.6)
Device wearing time, min/day, mean (SD)	1276.5 (129.0)	1306.6 (110.6)
Steps, steps/day, mean (SD)	8,799 (3344)	9,043 (3423)
Sedentary time, min/day, mean (SD)	640.5 (121.1)	658.0 (117.2)
LPA time, min/day, mean (SD)	271.1 (78.7)	271.6 (79.7)
MVPA time, min/day, mean (SD)	31.9 (30.7)	34.3 (30.3)
Season at recruitment		
Autumn, n (%)	396 (38.3)	279 (32.5)
Spring, n (%)	218 (21.1)	224 (26.1)
Summer, n (%)	192 (18.6)	349 (40.6)
Winter, n (%)	227 (22.0)	7 (0.8)

LPA, light-intensity physical activity; MVPA, moderate-to-vigorous-intensity physical activity; SD, standard deviation.

eTable 3. Characteristics of physical activity-associated variables for overall period

Month	Number of included participants, n	Age, years[a]	Proportion of women, %	Device wearing time, min/day[a]	Steps, steps/day[a]	LPA time, min/day[a]	MVPA time, min/day[a]
2022-09	62	49.6 (13.2)	24.2	1,166.7 (187.9)	8,912 (3,306)	277.5 (84.5)	31.1 (28.4)
2022-10	161	52.8 (14.0)	27.3	1,261.7 (144.9)	8,880 (2,784)	282.8 (79.0)	27.5 (26.6)
2022-11	224	54.0 (14.3)	30.4	1,253.8 (164.7)	8,623 (2,859)	273.5 (78.4)	26.2 (23.7)
2022-12	284	54.5 (14.0)	32.4	1,259.6 (157.1)	8,337 (2,865)	271.9 (81.8)	24.7 (23.1)
2023-01	332	55.6 (14.1)	34.6	1,262.8 (162.6)	8,214 (3,008)	259.7 (79.9)	26.8 (28.9)
2023-02	394	56.3 (14.4)	35.8	1,269.9 (154.3)	8,376 (3,116)	263.9 (81.5)	27.2 (25.4)
2023-03	501	56.9 (14.4)	35.5	1,251.6 (164.8)	8,769 (3,343)	268.6 (82.1)	30.4 (30.5)
2023-04	636	56.8 (14.4)	36.5	1,263.1 (162.2)	9,083 (3,470)	272.2 (80.2)	32.5 (31.6)
2023-05	799	57.9 (14.4)	36.7	1,271.3 (147.5)	9,019 (3,500)	270.8 (80.3)	33.9 (33.5)
2023-06	979	57.9 (14.2)	37.8	1,269.0 (152.7)	8,863 (3,529)	268.4 (83.6)	32.7 (31.2)
2023-07	1,123	57.2 (14.1)	37.8	1,244.9 (182.2)	8,439 (3,388)	261.7 (84.3)	32.5 (31.6)
2023-08	1,206	57.5 (14.0)	37.3	1,234.8 (187.6)	7,925 (3,276)	252.9 (83.2)	28.2 (27.4)
2023-09	1,340	58.2 (14.0)	36.9	1,252.0 (174.5)	8,214 (3,355)	253.4 (79.9)	31.2 (29.8)
2023-10	1,380	58.9 (13.8)	36.9	1,273.1 (154.6)	8,864 (3,495)	266.2 (79.2)	34.0 (32.7)
2023-11	1,438	59.4 (13.5)	37.1	1,282.0 (153.8)	8,883 (3,518)	266.4 (79.5)	34.6 (33.4)
2023-12	1,440	59.4 (13.4)	37.4	1,276.9 (161.6)	8,730 (3,519)	267.2 (81.9)	32.4 (31.1)
2024-01	1,373	59.4 (13.5)	37.5	1,280.2 (156.1)	8,275 (3,416)	251.8 (78.8)	30.3 (28.7)
2024-02	1,311	59.4 (13.4)	37.1	1,282.3 (159.1)	8,463 (3,485)	254.2 (77.9)	31.8 (30.6)
2024-03	1,248	59.4 (13.4)	37.4	1,278.1 (160.4)	8,610 (3,510)	258.6 (78.4)	32.2 (29.9)
2024-04	1,150	59.6 (13.2)	37.6	1,270.2 (166.1)	9,194 (3,599)	267.1 (79.9)	36.2 (32.3)
2024-05	1,027	59.7 (13.1)	37.6	1,264.9 (171.5)	9,082 (3,556)	266.0 (80.5)	35.2 (30.7)
2024-06	855	59.8 (12.9)	37.2	1,256.3 (179.9)	9,012 (3,554)	264.9 (81.6)	35.9 (32.5)
2024-07	664	60.2 (12.6)	36	1,255.0 (170.2)	8,615 (3,634)	261.1 (82.4)	33.7 (32.6)
2024-08	514	62.3 (12.0)	34.6	1,243.9 (193.8)	8,193 (3,520)	253.2 (84.1)	33.0 (33.1)
2024-09	379	62.8 (11.8)	33.2	1,278.0 (164.5)	8,454 (3,570)	258.3 (83.1)	34.9 (33.6)

2024-10	252	61.4 (11.4)	32.9	1,257.3 (197.4)	8,662 (3,310)	261.3 (79.3)	33.0 (25.4)
2024-11	135	60.1 (11.5)	32.6	1,265.2 (187.6)	8,882 (3,311)	264.4 (80.4)	34.6 (31.3)
2024-12	14	54.9 (12.4)	42.9	1,237.2 (219.4)	7,455 (2,938)	248.0 (76.7)	19.2 (22.0)

LPA, light-intensity physical activity; MVPA, moderate-to-vigorous-intensity physical activity.

[a] Data are presented as mean (standard deviation).

eTable 4. Characteristics of baseline sleep-associated variables divided by recruitment category

Variables	Recruitment category	
	Concurrent-visit (n=826)	Post-visit (n=733)
Age, years, mean (SD)	57.0 (14.0)	59.0 (13.1)
Age category		
20−39, n (%)	106 (12.8)	69 (9.4)
40−49, n (%)	213 (25.8)	141 (19.2)
50−59, n (%)	117 (14.2)	125 (17.1)
60−69, n (%)	165 (20.0)	201 (27.4)
70−79, n (%)	203 (24.6)	173 (23.6)
≥80, n (%)	22 (2.7)	24 (3.3)
Sex, men, n (%)	295 (35.7)	248 (33.8)
Time in bed, min/night, mean (SD)	429.3 (47.9)	423.9 (49.9)
Deep sleep time, min/night, mean (SD)	58.9 (13.4)	57.2 (12.7)
REM sleep time, min/night, mean (SD)	77.4 (18.0)	75.3 (18.7)
Light sleep time, min/night, mean (SD)	236.8 (35.8)	234.9 (36.7)
Total sleep time, min/night, mean (SD)	373.1 (41.2)	367.3 (43.0)
Sleep efficiency, %/night, mean (SD)	93.4 (5.0)	93.5 (3.4)
Season at recruitment		
Autumn, n (%)	325 (39.3)	249 (34.0)
Spring, n (%)	166 (20.1)	191 (26.1)
Summer, n (%)	153 (18.5)	287 (39.2)
Winter, n (%)	182 (22.0)	6 (0.8)

REM, rapid eye movement; SD, standard deviation.

eTable 5. Characteristics of sleep-associated variables for overall period

Month	Number of included participants, n	Age, years[a]	Proportion of men, %	Total sleep time, min/night[a]	Deep sleep time, min/night[a]	REM sleep time, min/night[a]	Light sleep time, min/night[a]
2022-09	33	51.4 (13.6)	24.2	367.9 (39.3)	60.7 (12.4)	79.9 (16.6)	227.3 (33.2)
2022-10	122	52.7 (13.9)	25.4	379.8 (45.9)	62.4 (14.3)	80.4 (17.1)	237.0 (38.9)
2022-11	183	54.3 (13.9)	26.2	380.5 (41.8)	61.0 (13.1)	81.1 (17.5)	238.4 (35.6)
2022-12	216	54.7 (13.8)	29.2	383.4 (39.6)	62.4 (11.8)	82.4 (16.7)	238.7 (33.6)
2023-01	260	55.6 (13.8)	31.2	383.9 (39.4)	61.2 (12.9)	83.2 (17.1)	239.5 (35.5)
2023-02	312	56.6 (14.1)	34	382.3 (41.2)	60.1 (12.7)	80.6 (18.1)	241.6 (38.3)
2023-03	382	56.4 (14.4)	34.3	374.7 (41.0)	59.2 (13.1)	78.2 (18.2)	237.3 (35.6)
2023-04	478	57.1 (14.2)	34.9	372.7 (40.2)	58.5 (13.0)	76.9 (18.8)	237.3 (35.9)
2023-05	601	57.6 (14.2)	35.1	369.0 (39.6)	57.6 (13.4)	76.2 (19.1)	235.2 (34.7)
2023-06	750	57.6 (13.8)	35.6	365.7 (39.6)	58.1 (13.2)	74.6 (18.4)	233.0 (33.9)
2023-07	870	56.8 (13.7)	34.7	360.4 (38.3)	58.1 (12.9)	73.0 (18.4)	229.3 (31.7)
2023-08	878	56.8 (13.8)	34.4	360.2 (39.2)	57.9 (12.9)	72.4 (18.7)	229.9 (32.7)
2023-09	1,026	57.6 (13.7)	34.6	364.7 (39.2)	57.8 (13.1)	73.3 (18.5)	233.6 (34.8)
2023-10	1,077	58.4 (13.6)	35.6	372.6 (41.0)	57.7 (13.2)	76.2 (18.1)	238.7 (36.0)
2023-11	1,172	59.2 (13.4)	35	377.5 (43.0)	57.7 (13.4)	77.6 (18.5)	242.3 (37.3)
2023-12	1,142	59.2 (13.1)	34.3	380.1 (43.9)	57.7 (13.3)	78.6 (18.3)	243.8 (37.7)
2024-01	1,052	59.0 (13.3)	34.1	384.1 (42.6)	58.3 (13.4)	79.2 (18.5)	246.6 (38.0)
2024-02	1,056	59.3 (13.2)	35.6	381.8 (43.8)	57.8 (13.7)	78.8 (18.6)	245.3 (37.4)
2024-03	976	59.1 (13.2)	35.2	377.7 (42.4)	57.7 (13.5)	77.4 (18.4)	242.6 (36.6)
2024-04	884	59.3 (13.0)	36.2	371.5 (42.3)	57.2 (13.4)	75.5 (18.6)	238.8 (35.8)
2024-05	789	59.5 (12.9)	35	367.0 (41.4)	57.1 (13.3)	74.4 (18.9)	235.5 (35.2)
2024-06	650	59.9 (12.8)	33.7	359.2 (41.2)	56.3 (13.5)	72.9 (18.7)	229.9 (34.0)
2024-07	509	59.7 (12.6)	32.6	355.5 (42.5)	56.2 (13.2)	70.5 (19.0)	228.8 (35.2)
2024-08	373	61.9 (11.6)	30	356.7 (40.8)	56.2 (13.3)	69.2 (18.2)	231.3 (35.0)
2024-09	307	62.7 (11.5)	30.9	363.3 (42.1)	55.1 (13.4)	70.3 (17.7)	237.9 (35.7)

2024-10	195	61.6 (10.8)	29.7	367.2 (44.3)	55.1 (14.3)	73.0 (17.9)	239.1 (37.1)
2024-11	108	59.9 (10.5)	30.6	376.8 (47.7)	54.8 (14.6)	76.7 (20.2)	245.3 (40.5)
2024-12	16	54.0 (12.5)	37.5	372.7 (67.1)	57.7 (22.5)	75.1 (19.4)	239.9 (56.8)

REM, rapid eye movement.

[a] Data are presented as mean (standard deviation).

eTable 6. Characteristics of the existing cohort data for the participants and non-participants of the present study

Variables	Cohort[a,b]	Participation status of the present study among potential target: Participants (n=1,996)	missing	Non-participants (n=5,444)	missing
**Demographic data**[c]					
Age, years, mean (SD)		57.6 (13.8)	0 (0.0)	60.2 (14.4)	0 (0.0)
Sex, men, n (%)		748 (37.5)	0 (0.0)	2,161 (39.7)	0 (0.0)
**Cognitive function data**[d]					
MMSE-J, points, mean (SD)	A	28.4 (1.8)	3 (0.2)	28.2 (1.9)	28 (0.5)
MoCA-J, points, mean (SD)	A	25.7 (2.9)	5 (0.3)	25.1 (3.1)	29 (0.5)
**Questionnaire data**					
Housing damage by GEJE (completely destroyed), n (%)	B	102 (5.3)	67 (3.4)	268 (5.1)	206 (3.8)
University or higher educational attainment, n (%)	B	842 (43.0)	38 (1.9)	1,938 (36.3)	110 (2.0)
Job with income, n (%)	C	864 (44.1)	38 (1.9)	2,069 (38.8)	121 (2.2)
Living alone, n (%)	C	126 (6.4)	35 (1.8)	340 (6.3)	110 (2.0)
CES-D, points, mean (SD)	C	10.8 (7.5)	183 (9.2)	10.6 (6.8)	610 (11.2)
LSNS, points, mean (SD)	C	15.3 (5.6)	73 (3.7)	15.5 (5.5)	227 (4.2)
Kessler 6, points, mean (SD)	C	3.0 (3.7)	107 (5.3)	2.7 (3.3)	352 (6.4)
Alcohol status	C		102 (5.1)		331 (6.1)
Current drinker, n (%)		1,090 (57.5)		2,798 (54.7)	
Former drinker, n (%)		52 (2.7)		129 (2.5)	
Smoking status	C		109 (5.5)		347 (6.4)
Current Smoker, n (%)		149 (7.9)		407 (8.0)	
Former smoker, n (%)		559 (29.6)		1,484 (29.1)	
Self-reported diabetes, n (%)	C	68 (3.5)	32 (1.6)	221 (4.1)	98 (1.8)
Self-reported hypertension, n (%)	C	350 (17.8)	32 (1.6)	1,070 (20.0)	98 (1.8)
Self-reported hyperlipidemia, n (%)	C	222 (11.3)	32 (1.6)	655 (12.3)	98 (1.8)
**Physiological data**					

Grip strength, kg, mean (SD)	C	31.9 (9.2)	94 (4.7)	31.7 (9.5)	265 (4.8)
BMI, kg/m^2, mean (SD)	C	23.0 (3.5)	84 (4.2)	22.9 (3.4)	361 (6.6)
SBP, mm Hg, mean (SD)	C	123.0 (17.4)	78 (3.9)	126.0 (18.4)	258 (4.7)
DBP, mm Hg, mean (SD)	C	76.6 (11.0)	78 (3.9)	77.0 (11.1)	258 (4.7)
**Laboratory data**					
HbA1c, %, mean (SD)	C	5.5 (0.4)	79 (3.9)	5.6 (0.5)	265 (4.8)
TG, mg/dL, mean (SD)	C	111.8 (76.7)	79 (3.9)	116.5 (81.0)	265 (4.8)
Total cholesterol, mg/dL, mean (SD)	C	204.5 (34.3)	79 (3.9)	205.8 (34.9)	265 (4.8)
LDL-cholesterol, mg/dL, mean (SD)	C	63.2 (16.8)	79 (3.9)	63.0 (16.6)	265 (4.8)
Creatinine, mg/dL, mean (SD)	C	0.7 (0.2)	79 (3.9)	0.7 (0.2)	265 (4.8)
AST, U/L, mean (SD)	C	21.7 (9.8)	79 (3.9)	22.3 (9.8)	265 (4.8)
ALT, U/L, mean (SD)	C	20.2 (14.2)	79 (3.9)	20.8 (13.7)	265 (4.8)

ALT, alanine aminotransferase; AST, aspartate aminotransferase; BMI, body mass index; CES-D, The Center for Epidemiologic Studies Depression Scale; DBP, diastolic blood pressure; GEJE, Great East Japan Earthquake; LDL, low-density lipoprotein; HbA1c, hemoglobin A1c; LSNS, Lubben Social Network Scale; MMSE-J, Japanese version of the Mini-Mental State Examination; MoCA-J, Japanese version of the Montreal Cognitive Assessment; SBP, systolic blood pressure; SD, standard deviation; TG, triglyceride.

[a] A: Secondary survey of the TMM Brain MRI Study (Oct 2019 to Mar 2024); B: Baseline survey of the TMM Comm Cohort or BirThree Cohort Study (Jul 2017 to Oct 2019); C: Secondary survey of the TMM Comm Cohort or BirThree Cohort Study (Jun 2017 to Mar 2021)

[b] Mean interval between A and C was 1306.8 (SD 429.8) days

[c] Age is at the time of participation of the secondary survey of the TMM Brain MRI Study

[d] Cognitive functions were assessed by trained staff

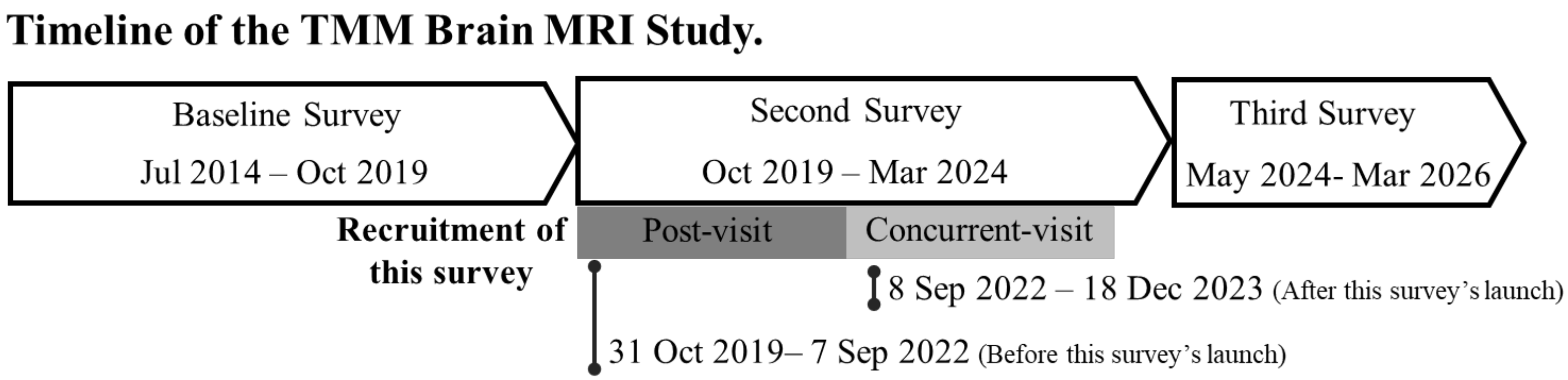

eFigure 1. Timeline of the TMM Brain MRI Study and recruitment for the present study

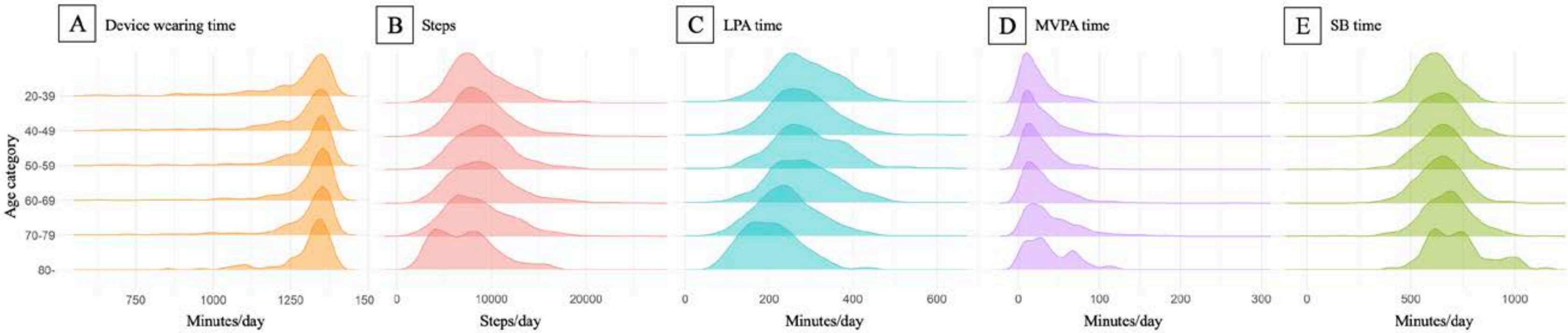

eFigure 2. Ridgeline plot for baseline physical activity-associated variables stratified by age category

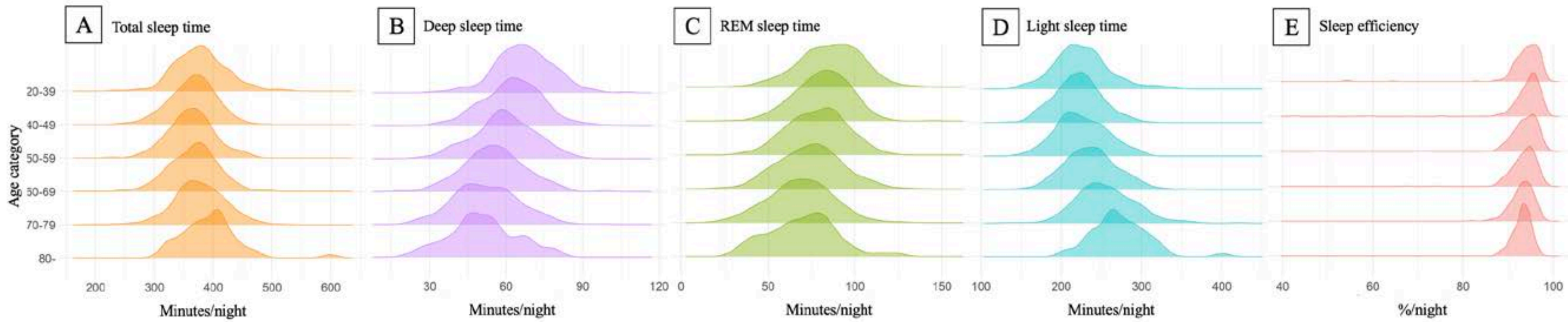

eFigure 3. Ridgeline plot for baseline sleep-associated variables stratified by age category
